# Supplementary material for: Efficiently Substituting Dietary Fish Meal with Terrestrial Compound Protein Enhances Growth, Health, and Protein Synthesis in Largemouth Bass
Source: Animals (Basel). 2024 Jul 28;14(15):2196. doi: 10.3390/ani14152196 (PMC11311014; doi:10.3390/ani14152196)
Supplement: Supplementary file 1 [file animals-14-02196-s001.zip › animals-3078431-supplementary.pdf]

Table S1 Primers used for detecting gene expression in this study

Genes	Forward primer(5′→3′)	Reverse Primer(3′→5′)
il-1β	AGCCGTCATTGAACATGGGA	GAAACATCAGGGGGTGACCA
il-6	CGCGCAATTTGCCGATGATA	CGTTGTTGCTGGTTGCATGA
tnf-α	TACAGCCAAGCGTCCTTCAG	GGACCAGCGCTGAACAGTAT
il-10	CCACCAGAATGACTCCTCGG	TGGTTGTTGCACATGGGACT
tgf-β1	CTTTACTACGTGGGCAGGCA	ATAGGTTTGAGGCGAGGCAG
zo-1	CCCCAACAAAAACAGAGCGG	GCTGCGAAGACCACGAAATC
claudin-3	AGAAGCAGTACAAGCCACCC	GGACACTCCATGGGCGTTTA
occludin	AGCGTCTTTAAAGCGGGGTC	TTGGAGCAGTTAACCCGACA
sirt1	TGGATTGTGAGGCTGTAAGG	ATGAGGAATGGAGTTTGGGA
pgc1-α	GGACGTGACCAATGCCAGTGA	ATAGCTGAGTTGGGAGTTTGCGG
pi3k	GGATGAGACACAGAAGATGCGA	CCTCAGGTTTCCCAGTTGGT
akt	TCTGGAGCATGTCTGCCAAT	TTTTTGCCAAAGGACAGCCG
mtor	CCAAAGACGTGCTGTTCACC	GAGCCTTCAGAAACCTGCGA
s6k1	GATTCTTGTGTGCGCCGTTT	ACCTACGGAGAAAGCAACCG
4e-bp1	ATATCCGATACAGGCGCGTT	TGGTCTTCTGGCAGTCAGTG
β-actin	ATCGTCCACCGCAAATGCTT	TGGTGTGGTTGTTTTGCACAG

Note: il-1β, interleukin 1β; il-6, interleukin 6; tnf-α: tumour necrosis factor α; il-10, interleukin 10; tgf-β, transforming growth factor β; mtor, target of rapamycin; pi3k, phosphoinositide 3-kinase; akt, protein kinase B; 4e-bp1, eukaryotic translation initiation factor 4E -binding protein 1; s6k1, ribosomal protein S6 kinase 1; zo-1, zonula occluden 1; sirt1, silent information regulator 1; pgc1-α, peroxisome proliferator-activated receptor-γ coactivator.

Table S2 Muscle textural properties and edible quality of largemouth bass fed with different diets

Items	Groups			
	T1	T2	T3	T4
**Proximate compositions**				
Moisture (%)	76.32 ± 0.57	75.71 ± 0.68	76.82 ± 0.79	76.55 ± 0.74
Crude protein (%)	84.75 ± 1.20b	85.17 ± 1.49b	87.01 ± 1.66ab	92.00 ± 1.73a
Crude lipid (%)	8.99 ± 0.10	8.81 ± 0.31	8.59 ± 0.85	7.45 ± 0.49
Glycogen (mg g-1)	0.13 ± 0.02a	0.06 ± 0.00b	0.06 ± 0.01b	0.06 ± 0.01b
AMP (nmol gprot-1)	424.35 ± 44.62	412.15 ± 12.94	381.44 ± 0.55	344.32 ± 24.80
ATP (μmol gprot-1)	115.94 ± 3.75	110.74 ± 4.47	111.83 ± 10.60	125.05 ± 2.51
ATP/AMP	273.22 ± 8.74b	268.69 ± 14.73b	293.18 ± 23.34ab	363.18 ± 13.05a
**Edible quality**				
CP (%)1	84.82 ± 0.36a	80.43 ± 2.42ab	75.52 ± 0.59b	77.61 ± 1.06b
WHC (%)2	5.57 ± 0.20	5.90 ± 0.39	6.07 ± 0.80	6.14 ± 0.60
**Textural properties**				
Hardness (gf)	77.14 ± 8.59	83.90 ± 4.10	81.83 ± 4.00	92.51 ± 9.95
Adhesiveness(gf)	0.55 ± 0.45	0.48 ± 0.01	0.41 ± 0.08	0.56 ± 0.34
Springiness(mm)	0.54 ± 0.01	0.49 ± 0.01	0.52 ± 0.02	0.5 ± 0.01
Chewiness(gf)	19.84 ± 3.68	24.28 ± 2.36	25.14 ± 3.41	29.16 ± 4.50
Gumminess (gf-mm)	49.36 ± 6.24	47.91 ± 3.90	46.74 ± 4.91	55.60 ± 7.51
Cohesiveness	0.64 ± 0.01	0.60 ± 0.01	0.63 ± 0.01	0.61 ± 0.01
Resilience	0.79 ± 0.03	0.72 ± 0.02	0.73 ± 0.02	0.75 ± 0.02
Tenderness (gf)	1417.48 ± 68.77	1479.98 ± 66.09	1534.42 ± 70.50	1616.9 ± 41.63

Note: Values are presented as the mean ± SEM (n = 4). Values in each row without sharing a common letter are significantly different ($P < 0.05$). 1Cooking percentage (CP) = 100 × [muscle initial weight before cooking (g) - muscle weight after cooking (g)]/muscle initial weight before cooking (g). 2Water holding capacity (WHC) = 100 × [muscle initial weight before extrusion (g) - muscle weight after extrusion (g)]/muscle initial weight before extrusion (g).

Table S3 Serum biochemical indicators of largemouth bass fed with different diets

Items	Groups			
	T1	T2	T3	T4
**Biochemical indicators**				
Total protein (mg mL-1)	45.63 ± 3.49	45.12 ± 0.89	46.09 ± 2.60	58.49 ± 7.45
Albumin (mg mL-1)	6.24 ± 0.45	8.10 ± 0.40	6.49 ± 0.84	7.04 ± 0.65
Globulin (mg mL-1)	41.55 ± 3.20	41.75 ± 1.88	41.20 ± 2.04	50.86 ± 6.64
Total amino acids (umol mL-1)	56.62 ± 3.21c	83.46 ± 1.78b	105.88 ± 11.41a	111.91 ± 4.10a
Serum ammonia (umol L-1)	338.33 ± 12.22b	784.26 ± 77.20a	373.98 ± 52.42b	463.26 ± 30.54b
Urea nitrogen (mmol L-1)	233.79 ± 17.20b	439.57 ± 37.97a	220.14 ± 11.43b	268.47 ± 6.99b
ALT (U L-1)	2.72 ± 0.89	1.10 ± 0.27	2.03 ± 0.69	2.26 ± 1.09
AST (U L-1)	16.28 ± 0.34a	8.68 ± 0.41b	10.31 ± 1.51b	11.48 ± 0.79b
ACP (U L-1)	13.57 ± 0.69	13.64 ± 0.80	14.54 ± 1.38	15.77 ± 1.40
ALP (U L-1)	13.70 ± 1.01	14.57 ± 1.26	14.36 ± 3.86	18.80 ± 1.53
**Antioxidant parameters**				
T-AOC (U mL-1 prot)	0.30 ± 0.01	0.31 ± 0.02	0.32 ± 0.03	0.36 ± 0.01
MDA (nmoL mL-1 prot)	35.23 ± 2.31a	39.31 ± 3.91a	33.28 ± 0.82ab	29.00 ± 2.49b

Note: Values are presented as the mean ± SEM (n = 4). Values in each row without sharing a common letter are significantly different ($P < 0.05$).

Table S4 Intestinal biochemical indicators of largemouth bass fed with different diets

Items	Groups			
	T1	T2	T3	T4
**Digestive enzymes**				
Trypsin (U g-1 prot)	3.43 ± 0.08	3.90 ± 0.52	4.43 ± 0.32	4.45 ± 0.77
Lipase (U g-1 prot)	3.57 ± 0.19b	5.24 ± 0.53a	5.3 ± 0.37a	6.24 ± 0.12a
Amylase (U mg-1 prot)	0.08 ± 0.02	0.09 ± 0.00	0.08 ± 0.01	0.07 ± 0.01
**Health indicators**				
ACP (U g-1prot)	42.05 ± 3.00	37.04 ± 1.93	46.03 ± 3.69	39.89 ± 2.36
ALP (U g-1prot)	72.75 ± 3.69b	86.32 ± 3.91b	79.47 ± 9.93b	162.2 ± 6.53a
T-AOC (U mg-1 prot)	5.64 ± 0.45	4.78 ± 0.13	5.08 ± 0.11	4.57 ± 0.57
MDA (nmoL mg-1 prot)	62.21 ± 3.10	48.62 ± 3.90	42.31 ± 17.16	48.62 ± 3.90

Note: MDA:malondialdehyde; SOD:Superoxide dismutase; CAT: Catalase; GSH-PX: glutathione peroxidase; ACP: acid phosphatase; ALP: alkaline phosphatase. Note: Values are presented as the mean ± SEM (n = 4). Values in each row without sharing a common letter are significantly different (P < 0.05).
